# Ultrastructure of the Dentin Pellicle and the Impact of Erosion

**DOI:** 10.1159/000527775

**Published:** 2022-10-28

**Authors:** Anton Schestakow, Christina Bauer, Matthias Hannig

**Affiliations:** Clinic of Operative Dentistry, Periodontology and Preventive Dentistry University Hospital, Saarland University, Homburg, Germany

**Keywords:** Demineralization, Dentin, Erosion, Salivary pellicle, Transmission electron microscopy

## Abstract

While the ultrastructure of the enamel pellicle and its erosion protective properties are well studied, the dentin pellicle is still neglected in dental research. Therefore, the ultrastructure and erosion protective properties of a pellicle formed on bovine dentin specimens were investigated in the present study. The dentin pellicle was formed in situ for 3, 30, 120, and 360 min at buccal or palatal oral sites of 3 subjects and analyzed by transmission electron microscopy. In order to clarify the impact of an erosive challenge to the ultrastructure of the pellicle and the underlying dentin, specimens were exposed to the oral cavity and eroded in vivo with 0.1% or 1% citric acid either immediately or after 30 min of pellicle formation. Specimens that were eroded without exposure to the oral cavity served as control. In another trial, specimens with a 30-min pellicle were exposed to the oral cavity for a further 60 min after the erosive challenge to investigate the effect of saliva on the impaired pellicle and dentin. Transmission electron micrographs reveal a globular and granular structured pellicle layer, which was thicker when the pellicle was formed buccally or with longer formation times. Erosion with citric acid reduced the thickness of the pellicle and interrupted its continuity. The dentin was also affected by erosion, which was represented by a lower electron density and formation of demineralized lacunae. These were infiltrated by a granular structured material when specimens were exposed to the oral cavity. After further intraoral exposure, the infiltration was more pronounced, indicating a significant impact of saliva on the demineralized dentin. A reformation of the dentin pellicle on the other hand did not occur. In conclusion, the dentin pellicle is neither acid-resistant nor able to effectively protect dentin from erosion.

## Introduction

Dental erosion is defined as a loss of dental hard tissue caused by nonbacterial acids [Imfeld, 1996]. When acids come into direct contact with the dental hard tissue, calcium and phosphate ions are dissolved from the inorganic matrix. In contrast to the predominantly inorganic enamel, the erosive process is more complicated in dentin due to the high organic content. Erosion has a high prevalence and is also clinically relevant. As the number and age of dentulous adults increases, erosive damage to teeth accumulates and leads to exposure of dentin [Jaeggi and Lussi, 2014; McKenna et al., 2020].

The erosive process is modulated by multiple behavioral and biological factors, of which human saliva is considered the most important host defense against erosion. Saliva contributes to the clearance of erosive substances from the oral cavity and provides buffering properties, thus neutralizing acids [Buzalaf et al., 2012]. Thanks to its calcium, phosphate, and fluoride content, saliva can also remineralize eroded surfaces [Amaechi and Higham, 2001]. In addition, saliva is rich in proteins that can adhere to teeth, forming a protective layer termed pellicle. The pellicle acts as a semipermeable membrane, reducing both the contact of acids with dental surfaces and the loss of ions. The pellicle itself has also buffering properties and can accumulate ions, providing a mineral depot for remineralization [Hannig and Hannig, 2014].

So far, the pellicle and its erosion protective properties have been intensively investigated on enamel, while the other dental hard tissue, dentin, is often neglected [Hannig and Hannig, 2014; Rasputnis et al., 2021]. Previous studies on the structure of the dentin pellicle show similarities to the pellicle formed on enamel [Rasputnis et al., 2021]. A pellicle formed on polished dentin specimens for 120 min consists of a 30–60 nm thick dense basal layer covered by a 300–750 nm thick loose and globularly structured outer layer [Hannig et al., 2007]. No further ultrastructural studies on the dentin pellicle are available to date. The literature on the erosion protective properties of the dentin pellicle is also scarce and controversial. Of the different measurements for erosion, e.g., measurement of calcium release or microhardness, only one qualitative measurement is available for the dentin pellicle [Rasputnis et al., 2021]. In that study, when dentin specimens with a 120-min pellicle were eroded extraorally with hydrochloric acid for 5 min, similar demineralization was observed on specimens with and without a pellicle. The pellicle was partially destroyed by erosion, suggesting that the dentin pellicle has limited erosion protective properties [Hannig et al., 2007].

Due to the few ultrastructural analyses and the controversially discussed erosion protective properties, the dentin pellicle merits further investigation. In previous ultrastructural studies, detailed characterizations of the dentin pellicle after different formation times are missing. The erosion studies were also limited by the extraoral application of the erosive agents since the impact of saliva was not recorded.

The aim of the present study was therefore to investigate the progress of pellicle formation on dentin specimens in situ. In order to analyze the erosion protective properties of the dentin pellicle, specimens were additionally eroded by rinsing the oral cavity with citric acid. The impact of saliva on the impaired pellicle and demineralized dentin was also studied by continuous intraoral exposure of specimens after the erosive challenge.

## Materials and Methods

### Subjects

Three volunteers (aged from 28 to 32 years) with a complete dentition and without active caries lesions, periodontitis, orthodontic appliances, or diseases of the oral mucosa and salivary glands participated in the present study. They did not take any drugs or medication. Subjects have given their written informed consent, and the study protocol was approved by the Medical Ethics Committee of the Medical Association of Saarland (238/03-2012).

### Specimens and Splints

Specimens (*n* = 144) were made from lower incisors of 2-year-old calves from the slaughterhouse in Zweibrücken, Germany. The root of the teeth was separated into several parts, followed by removal of the cementum and circumpulpal dentin by wet grinding. After they were scored in the outer third of the dentin, they were stored in 0.1% thymol until use. Shortly before the trial, specimens were disinfected by ultrasonication for 5 min in 70% ethanol. Then, they were fractured along the score line in order to generate a surface of approximately 2 mm^2^ without a smear layer and with open dentinal tubules. For each trial, specimens were fixed with silicone impression material (PRESIDENT light body, Coltène/Whaledent GmbH + Co. KG, Langenau, Germany) to individual upper splints made from methacrylate (DURAN®, Scheu Dental GmbH, Iserlohn, Germany) in the region of the first upper molars and stored for 24 h in distilled water for rehydration.

### Pellicle Formation in situ

The present study consisted of 3 different trials starting between 8:00 and 9:00 a.m. after the subjects had brushed their teeth with a disposable toothbrush without dentifrice. In trial 1, a total of 8 specimens were fixed to the splints, with 4 specimens each in the first and second quadrant and 2 specimens each buccal and palatal. The subjects carried the splints intraorally for 3, 30, 120, and 360 min. The consumption of food and beverages was not allowed except for the 360 min of pellicle formation. In this case, the splints were stored in a humid chamber and reinserted after the teeth were cleaned, as described above. In trial 2, specimens were only fixed buccally and subjects rinsed for 1 min with 10 mL of 0.1% or 1% citric acid (pH 2.74; 2.36) either immediately or after 30 min of intraoral exposure. Specimens without a pellicle that were exposed to 50 mL citric acid in vitro served as control. After 30 min of pellicle formation followed by erosion, specimens were worn for a further 60 min in trial 3 in order to investigate the impact of saliva on the impaired pellicle and dentin.

### Transmission Electron Microscopy

After in vitro erosion or after specimens have been removed from the oral cavity, they were rinsed with sterile water and fixed with glutaraldehyde for 2 h. Then, specimens were washed in phosphate buffer, postfixed in 2% osmium tetroxide for 2 h, and washed in phosphate buffer again. The specimens were embedded in Araldite CY 212 (Serva, Heidelberg, Germany), and ultrathin sections were cut using an Ultracut E ultra-microtome (Reichert, Bensheim, Germany). Finally, the samples were stained with uranyl acetate and lead citrate and analyzed with a transmission electron microscope (TEM Tecnai 12 BioTwin, FEI Co., Eindhoven, The Netherlands) at a magnification of up to 100,000-fold.

## Results

### Trial 1

**Fig. 1:**
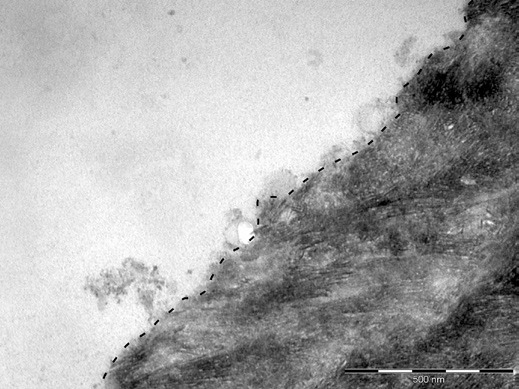
In situ pellicle formed over a period of 3 min at the buccal site (trial 1). Dentin was covered by a thin granular pellicle layer. The interface between the pellicle and dentin is marked by a dashed line. Original magnification: 70,000-fold.

**Fig. 2:**
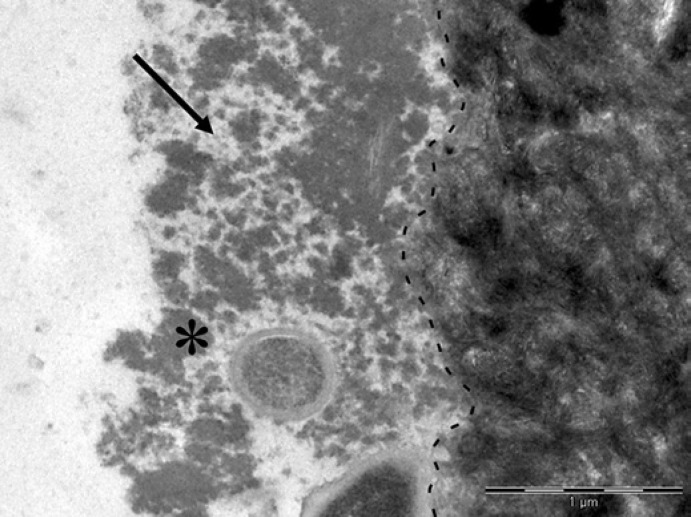
In situ pellicle formed over a period of 360 min at the buccal site (trial 1). Dentin was covered by a granularly (arrow) and globularly (*) structured pellicle layer. The interface between the pellicle and dentin is marked by a dashed line. Original magnification: 30,000-fold.

With the method presented here, dentin specimens were produced that had no smear layer. After 3 min of intraoral exposure, dentin specimens were only partially covered by a pellicle, regardless of the localization in the oral cavity. The pellicle had a homogeneous granular structure with few up to 300-nm thick adherent agglomerates (Fig. 1). As with longer pellicle formation times, the base layer was not detectable due to the electron density of the underlying dentin (online suppl. materials). The dentin matrix is dominated by numerous densely packed collagen fibrils with a striped pattern. When specimens were exposed to the oral cavity buccally for 30 min, the dentin was covered by a continuous pellicle with an average thickness of 200 nm (Table 1). Occasionally, the thickness exceeded 1 µm. In contrast to the buccal pellicle, the palatal pellicle was discontinuous so that an average thickness could not be determined. After 120 min of intraoral exposure, the overall thickness of the pellicle increased. The buccal pellicle was continuous and 450 nm thick, while the palatal pellicle was discontinuous. The pellicle thickness continued to increase and reached 1 µm and 200 nm, buccally and palatally, after 360 min of intraoral exposure, respectively. The heterogeneous structure of the pellicle consisted of both granular parts and up to 500-nm thick globular agglomerates, which were often separated by hollow spaces (Fig. 2). These agglomerates were primarily found at buccally formed pellicles. The number of adherent bacteria increased with the duration of intraoral exposure. While bacteria were rarely found after few minutes of pellicle formation, adherent bacteria and bacterial accumulation appeared more frequently after 360 min. Considering the intraoral localization of specimens, fewer bacteria adhered to the palatally formed pellicle.

### Trial 2

**Fig. 3:**
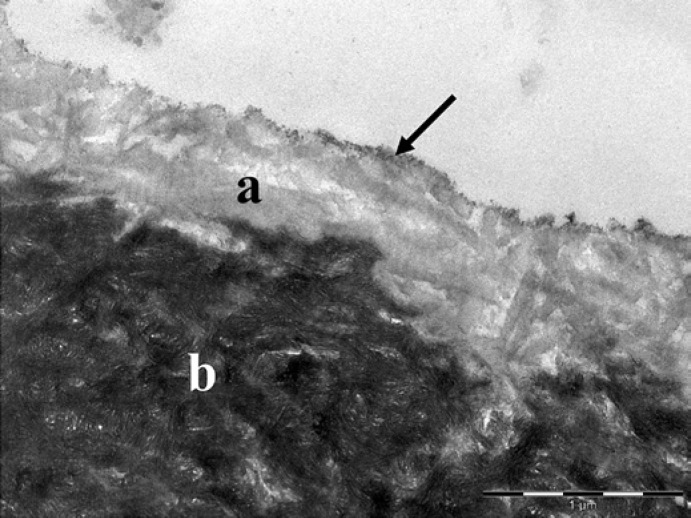
In vitro erosion of dentin with 1% citric acid (trial 2). The demineralized dentin (**a**) had a lower electron density than the unaffected dentin (**b**). Note the exposed collagen fibrils with a striped pattern and the electron-dense particles (arrow) at the surface. Original magnification: 30,000-fold.

Erosion of dentin specimens with 0.1% or 1% citric acid in vitro resulted in 150 nm or 700-nm deep demineralization, respectively. The demineralized dentin was characterized by exposed collagen fibrils and intervening hollow spaces (Fig. 3). Due to its lower electron density, the demineralized dentin could be clearly differentiated from the unaffected dentin. At the intermediate zone, fine electron-dense particles appeared representing disclosed hydroxyapatite crystals. The surface of the demineralized dentin was also covered by fine electron-dense particles.

When specimens were eroded in vivo with 0.1% or 1% citric acid immediately after insertion of the splints, demineralization of up to 450 nm or 2 µm was observed, respectively. The surface was again covered by electron-dense particles and, in addition, by a granularly and globularly structured layer. A similar granular structure infiltrated the hollow spaces, resulting in a dense appearance of the demineralized dentin.

**Fig. 4:**
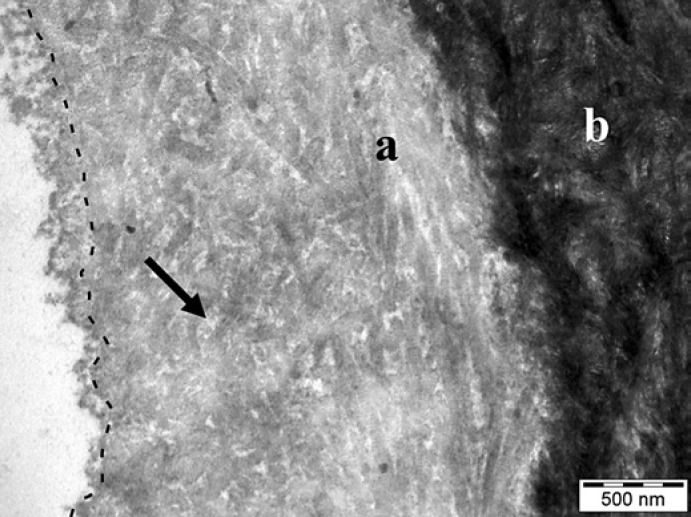
Dentin with a 30-min pellicle formed at the buccal site and eroded with 1% citric acid in vivo (trial 2). The demineralized dentin (**a**) had a lower electron density than the unaffected dentin (**b**) and was covered by a granular and globular structured pellicle layer. The interface between the pellicle and dentin is marked by a dashed line. Note the granular material infiltrating the hollow spaces of the demineralized dentin (arrow). Original magnification: 30,000-fold.

In contrast, when specimens were exposed to the oral cavity for 30 min, a pellicle was able to form before the erosive challenge. Rinsing with 0.1% citric acid induced a 400 nm deep demineralization in pellicle-covered specimens, which was also characterized by exposed collagen fibrils and a lower electron density of the dentin. The erosive challenge reduced the pellicle thickness to an average of 100–200 nm, and at the same time led to structural alterations such as discontinuities and loosening of the pellicle layer. In areas where loosening occurred, pellicle thickness of more than 2 µm could be measured. The alterations of the pellicle and underlying dentin were more pronounced when subjects rinsed with 1% citric acid. The pellicle layer had an average thickness of 160 nm and was more frequently interrupted in its continuity or even detached from the demineralized dentin. In contrast to the pellicle, which showed variations in ultrastructure, the demineralization zone had a uniform thickness. The demineralization was 1.3-µm deep and could be clearly differentiated from the unaffected dentin, which still showed a high electron density. The density of the demineralized dentin was partially higher on specimens covered by a 30-min pellicle than on specimens that were not exposed to the oral cavity, again due to infiltration by a granular material (Fig. 4).

### Trial 3

**Fig. 5:**
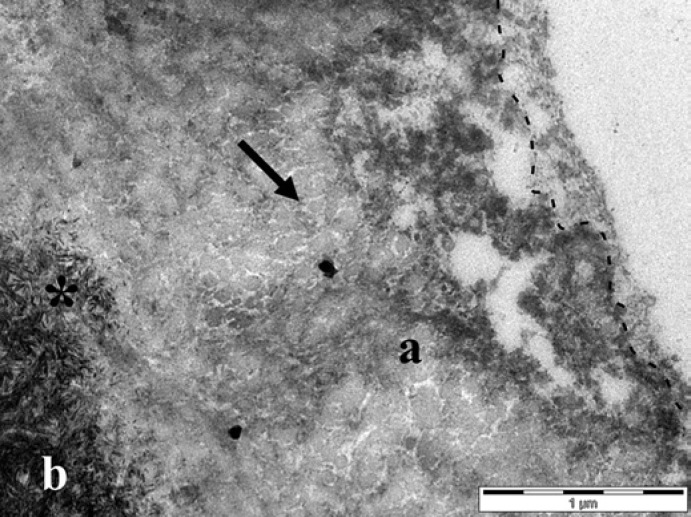
Dentin was exposed to the oral cavity for 30 min, eroded with 1% citric acid in vivo, and worn for a further 60 min (trial 3). The demineralized dentin (**a**) had a lower electron density than the unaffected dentin (**b**) and was covered by a granular and globular structured pellicle layer. The interface between the pellicle and dentin is marked by a dashed line. Note the granular material infiltrating the hollow spaces of the demineralized dentin (arrow) and the disclosed hydroxyapatite crystals at the intermediate zone (*). Original magnification: 30,000-fold.

After specimens remained in the oral cavity for a further 60 min following the erosion with 0.1% citric acid, a demineralization of 400 nm was still detectable. The pellicle had a granular structure and varied in thickness, being up to 2-µm thick at areas where loosening of the pellicle structure occurred. When erosion was performed with 1% citric acid, the demineralization was more pronounced with 2.5 µm on average. The pellicle was also granular, but the continuity was more frequently disrupted. The demineralization zone was infiltrated by a granular material and appeared denser than in specimens that were removed immediately from the oral cavity after erosion (Fig. 5).

## Discussion

The thickness of the pellicle increased during intraoral exposure, with the buccal pellicle being distinctly thicker than the palatally formed pellicle. With longer duration of pellicle formation, the number of globular structures and adherent bacteria increased. While the unaffected dentin showed a high electron density, rinsing with citric acid resulted in demineralization of the dentin and alterations of the pellicle, with the alterations being more pronounced when the higher concentrated acid was used. The demineralized dentin was infiltrated by a granular material on specimens that were exposed to the oral cavity. With further intraoral exposure, the demineralized dentin was even more affected by the infiltration, but the pellicle was not able to reform.

There are only few ultrastructural studies on the dentin pellicle in the literature. Hannig et al. [2007] and Abbas et al. [1985] examined a 120-min pellicle formed on dentin specimens at buccal or labial oral sites, respectively [Abbas et al., 1985; Hannig et al., 2007]. While Abbas et al. [1985] reported only of a thin film of varying thickness, Hannig et al. [2007] showed that the pellicle consists of a 30–60-nm thick electron-dense basal layer covered by a 300–750-nm thick loosely arranged and globularly structured outer layer [Abbas et al., 1985; Hannig et al., 2007]. In another study by Jung et al. [2010], dentin specimens were exposed to the oral cavity for 30–360 min and as a result, a pellicle has formed. However, the topic was not the dentin pellicle but the initial bacterial adherence [Jung et al., 2010]. Therefore, in the present study, the dentin pellicle was examined both buccally and palatally after different periods of pellicle formation. When dentin specimens were exposed for 3 min to the oral cavity, a thin granular layer could be detected by TEM. An electron-dense basal layer, which forms within a few minutes on enamel [Hannig et al., 2005; Hannig and Joiner, 2006; Joiner et al., 2008], was also reported for dentin [Hannig et al., 2007; Jung et al., 2010] and was not found in the present study. In contrast to the present study, Hannig et al. [2007] and Jung et al. [2010] formed the pellicle on polished and EDTA-treated dentin specimens [Hannig et al., 2007; Jung et al., 2010]. The modification of the dentin surface by EDTA and thus the pellicle formation cannot be excluded [Gandolfi et al., 2018]. Dentin itself has a high electron density that can also hinder the detection of an electron-dense basal layer. However, even after the disclosure of the pellicle by demineralization of dentin in trial 2, an electron-dense basal layer could not be detected. With regard to the different composition and morphology of the enamel and dentin [Low et al., 2008], this finding indicates a substrate-dependent initiation of pellicle formation. With longer pellicle formation times, the thickness of the pellicle increased in a time-dependent manner, reaching 1 μm or 200 nm after 360 min at buccal or palatal intraoral sites, respectively. Buccal specimens were exposed to saliva originating primarily from the parotid gland. Considering that the parotid gland contains protein aggregates [Rykke et al., 1997], and these were also found in the buccal pellicle, the rapid increase in pellicle thickness may be the result of deposition of protein aggregates rather than single proteins [Hannig, 1999]. Furthermore, the buccal pellicle is not exposed to the shearing forces of the tongue, which limits pellicle formation at palatal oral sites [Sonju-Clasen, 2000]. With longer pellicle formation duration, not only the thickness but also the number of adherent bacteria increased. In accordance with the literature, the first bacteria were detected after the first few minutes of pellicle formation [Jung et al., 2010]. When comparing the buccal and palatal pellicle, there were a site-dependent number of bacteria. Fewer bacteria adhered to the palatal pellicle, which is associated with the presence of lysozyme, an antibacterial protein that is 3 times more abundant in sublingual and submandibular glands than in the parotid gland [Hannig et al., 2005].

When buccal specimens with a 30-min pellicle were subjected to erosion, several ultrastructural alterations appeared in both the dentin and the pellicle. After exposure of the pellicle to 0.1% and especially 1% citric acid, the thickness decreased on average, with the pellicle being occasionally loosened, destroyed, or detached. While the duration of pellicle formation does not play a significant role in anti-erosive properties of the enamel pellicle [Hannig et al., 2003; Hannig et al., 2004], it does affect the acid resistance of the pellicle itself [Hannig et al., 2003]. In the study by Hannig et al. [2003], when the pellicle was formed on enamel for 2 and up to 24 h, exposure to acid resulted in interruptions especially of the 2-h pellicle layer [Hannig et al., 2003]. In the present study on the other hand, the 30-min pellicle showed not only interruptions in its continuity but also ultrastructural alterations, which can be attributed to the short duration of pellicle formation. With regard to demineralization of dentin specimens, even the intact pellicle did not prevent erosion, as in the study by Hannig et al. [2007]. The demineralization was characterized by a lower electron density and exposure of collagen fibrils. The thickness of the demineralization zone was not affected by the presence of a covering pellicle layer. Different aspects can be considered for the absent erosion protective properties of the dentin pellicle. The present TEM results indicate that the dentin pellicle is a highly porous network of proteins that is permeable for both, citric acid and ions. Hara et al. [2006] suggested that the pellicle is not able to protect dentin due to its higher solubility and faster demineralization compared to enamel [Hara et al., 2006]. In addition, the composition of the pellicle may have an impact on its anti-erosive properties [Hannig and Hannig, 2014], but there are only few studies on the composition of the dentin pellicle; so its role in the erosion process cannot be assessed [Rasputnis et al., 2021]. In this regard, the origin of specimens must also be considered. Due to chemical, physical, and morphological characteristics, bovine dentin is not only differently susceptible to erosion than human dentin [Wegehaupt et al., 2008; Yassen et al., 2011] but also affect pellicle formation. In the study by Pelá et al. [2018], the protein composition of a 120-min pellicle formed on bovine and human enamel was investigated. While several proteins were similar, some proteins were found exclusively in the pellicle on bovine or human enamel [Pelá et al., 2018]. According to a study on natural teeth of patients with gastroesophageal reflux disease, where the composition of the pellicle differed from patients with or without erosive lesions, the composition may play a role in the erosion protective properties of the pellicle [Martini, 2017]. However, in an in vitro study by Hove et al. [2007], no differences in calcium loss between pellicle-covered bovine and human enamel were recorded after an erosive treatment [Hove et al., 2007]. To what extent the difference in the composition of the pellicle is relevant for bovine and human dentin, merit further investigation.

When comparing the different erosion conditions of the present study, an infiltration of the demineralized dentin by a granular material was detected in specimens that were exposed to the oral cavity. A similar observation was made on enamel [Hannig et al., 2009]. In the study by Hannig et al. [2009], enamel specimens with a 120-min pellicle were demineralized by the application of acidic beverages. The demineralized lacunes were filled by a granular material indicating the formation of a subsurface pellicle [Hannig et al., 2009]. In the present study, specimens that were eroded immediately after insertion of the splints were also affected by this phenomenon. We suggest that during and after erosion the demineralized dentin is rapidly infiltrated with proteins not only from the pellicle but also from saliva. According to the study by Delecrode et al. [2015], the dentin pellicle contains proteins with the ability to interact with other proteins [Delecrode et al., 2015]. Therefore, after infiltration into the demineralized dentin, proteins could interact with the exposed collagen network. When the splints were worn for a further 60 min, an unreasonably deep demineralization was detected when 1% citric acid was used. Regarding the low number of subjects, this result might be a biological variance. The demineralized dentin, however, was even more densely infiltrated, indicating a “reparation” of the erosive damage by replacement with organic material. A deposition of minerals as in the in vitro study by Eisenburger et al. [2001] was not observed [Eisenburger et al., 2001], indicating that infiltration by organic material can hinder a potential deposition of minerals. The preventive strategy against dentin erosion with remineralizing agents must be critically reviewed, and new preventive approaches such as stabilization of the collagen network must be considered. The pellicle was not able to reform by further contact to saliva. In an in vitro study by Houghton et al. [2020], acid exposure reduced the protein content of an enamel pellicle, which could hardly be restored by reincubation in saliva [Houghton et al., 2020]. Together with the ultrastructural alterations of the dentin pellicle observed in the present study, protein adsorption and pellicle formation seem to proceed differently on an impaired pellicle. Furthermore, the previous erosion led to demineralization and exposure of collagenic fibrils of the dentin. It is known from enamel that pellicle formation is initiated by adsorption of salivary proteins to the inorganic matrix [Hannig and Hannig, 2014]. Pellicle formation on dentin might be hindered, when the inorganic matrix is lost after erosion.

The results of the present study underline the site-specific ultrastructure of a pellicle formed on dentin. Transmission electron micrographs indicate that the 30-min pellicle is not able to protect dentin from erosion, which is in contrast to data of the enamel pellicle. However, the demineralized dentin was rapidly infiltrated by a granular structure, which might represent salivary proteins or proteins released from the pellicle.

## Statement of Ethics

This study protocol was reviewed and approved by the Medical Ethics Committee of the Medical Association of Saarland (238/03-2012). Written informed consent was obtained from participants to participate in the study.

## Conflict of Interest Statement

The authors have no conflicts of interest to declare.

## Funding Sources

The study was supported by funding from the German Research Foundation (DFG, SFB 1027).

## Author Contributions

Anton Schestakow contributed to interpretation and drafted the manuscript. Christina Bauer contributed to data acquisition and interpretation and drafted the manuscript. Matthias Hannig contributed to conception, design, interpretation, and critically revised the manuscript. All authors gave their final approval and agree to be accountable for all aspects of the work.

## Data Availability Statement

Data generated or analyzed during this study are included in this article and its supplementary material files. Further data are available from the corresponding author. The results of this study are part of the thesis by Bauer (2011) published at Saarland University and State Library (doi:10.22028/D291-21553). We have the permission to republish the data.

## Supplementary Material

Supplementary data

Supplementary data

Supplementary data

Supplementary data

Supplementary data

Supplementary data

Supplementary data

Supplementary data

Supplementary data

Supplementary data

Supplementary data

Supplementary data

Supplementary data

Supplementary data

Supplementary data

Supplementary data

Supplementary data

## Figures and Tables

**Table 1:** Measurable characteristics of the pellicle and demineralized dentin

Trial	Erosion	Citric acid, %	Pellicle	Demineralization
1 (30 min)	None		200 nm	none

2	In vitro	0.1	None	150 nm
	
		1	None	700 nm
	
	In vivo, immediately	0.1	Discontinuous	450 nm
	
		1	Discontinuous	2 µm
	
	In vivo, after 30 min	0.1	100–200 nm	400 nm
	
		1	160 nm	1.3 pm

3	In vivo, after 30 min, further 60 min of intraoral exposure	0.1	15–150 nm	400 nm
	
		1	200 nm	2.5 pm
