# Supplementary figures and images for: Ultrastructure of the Dentin Pellicle and the Impact of Erosion
Source: Caries Res. 2022 Oct 28;56(5-6):488–95. doi: 10.1159/000527775 (PMC9932831; doi:10.1159/000527775)

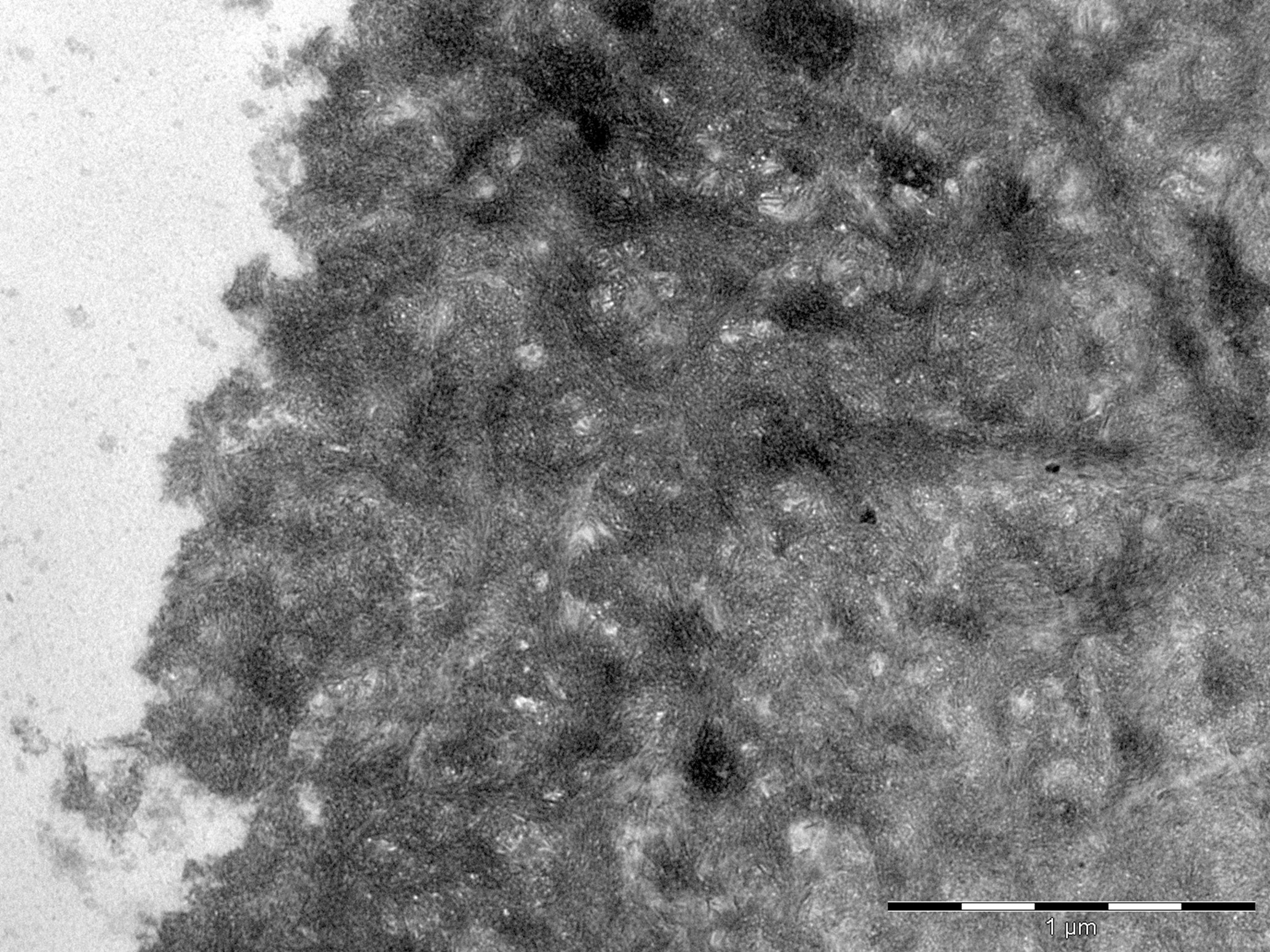

Supplement: Supplementary file 1 — Supplementary data [file cre-0056-0488-s01.jpg]

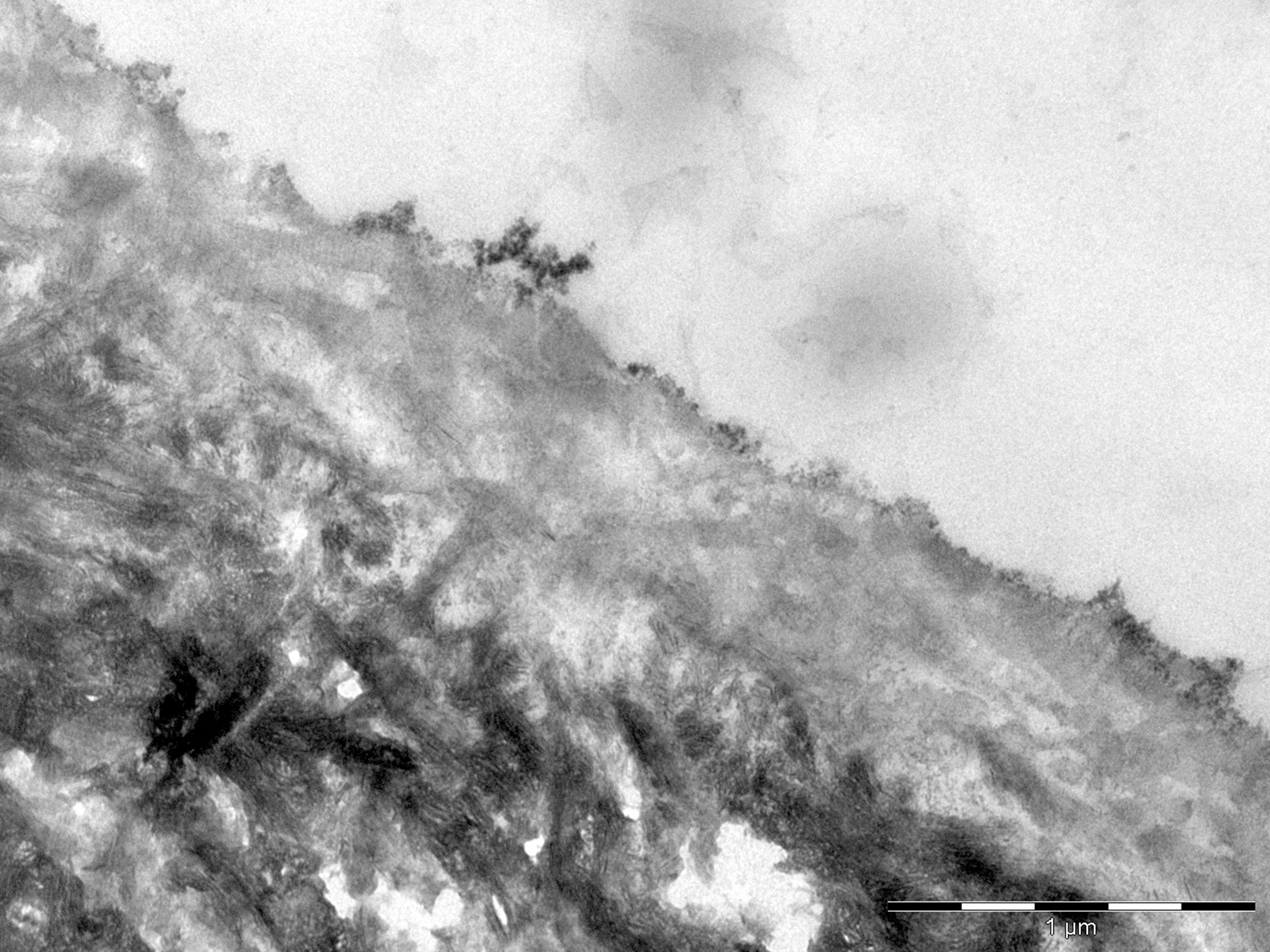

Supplement: Supplementary file 2 — Supplementary data [file cre-0056-0488-s02.jpg]

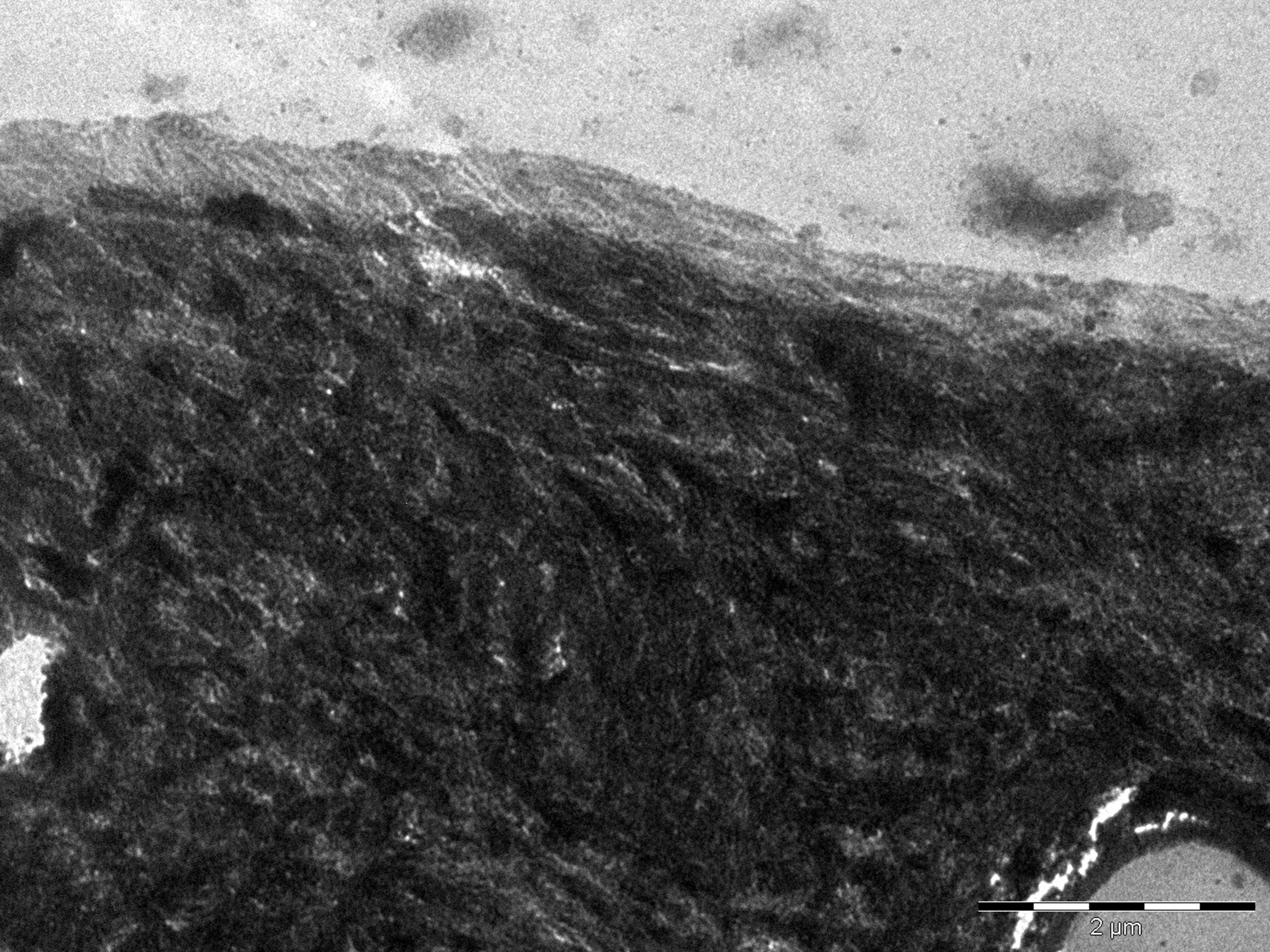

Supplement: Supplementary file 3 — Supplementary data [file cre-0056-0488-s03.jpg]

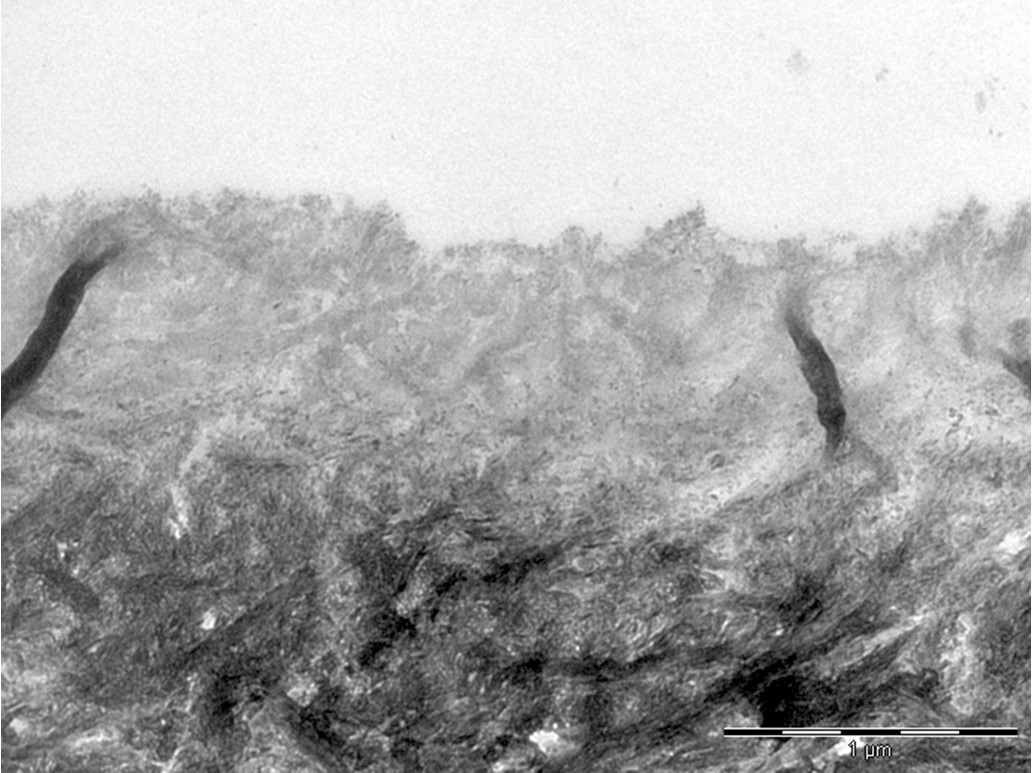

Supplement: Supplementary file 4 — Supplementary data [file cre-0056-0488-s04.jpg]

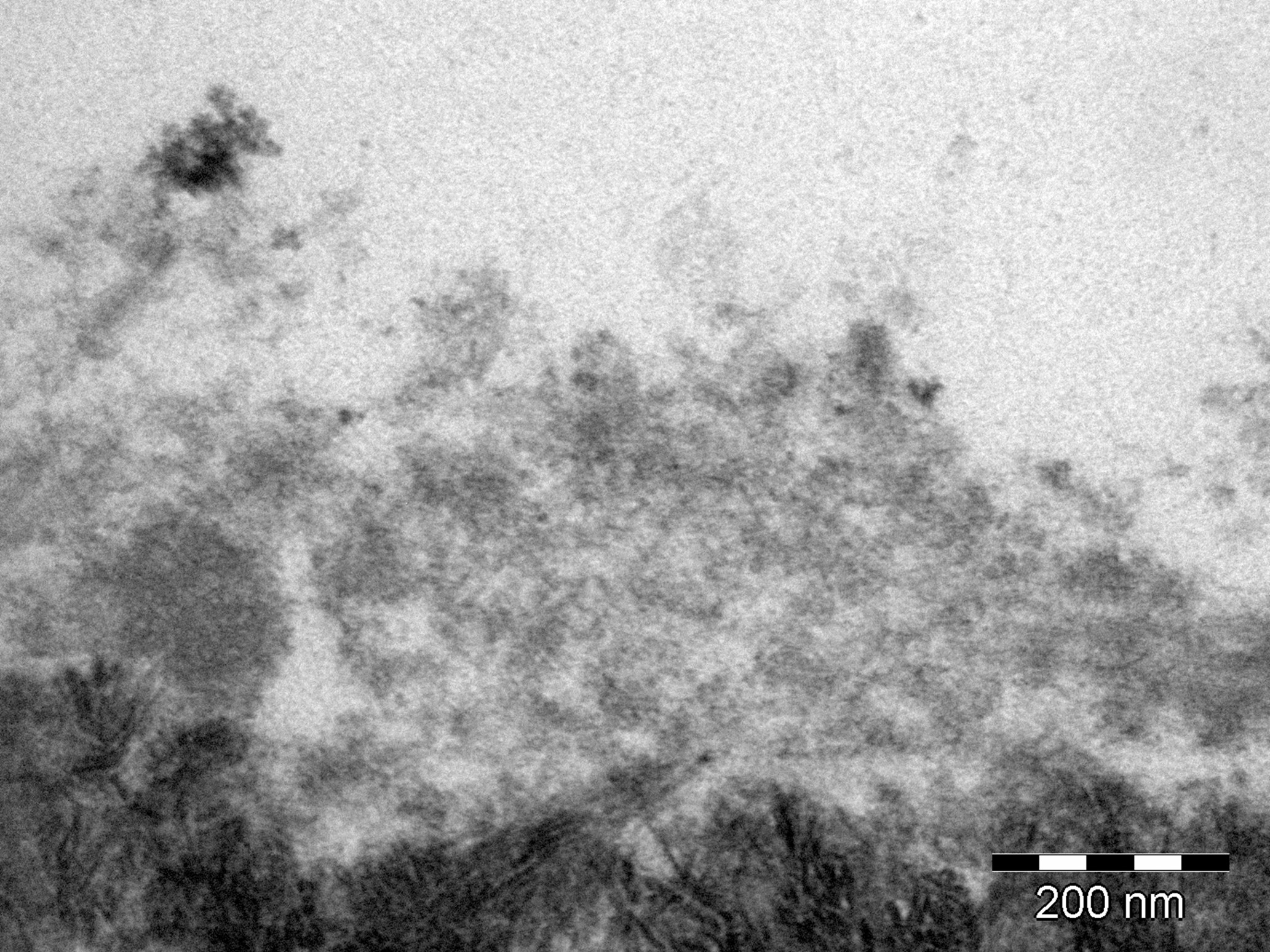

Supplement: Supplementary file 5 — Supplementary data [file cre-0056-0488-s05.jpg]

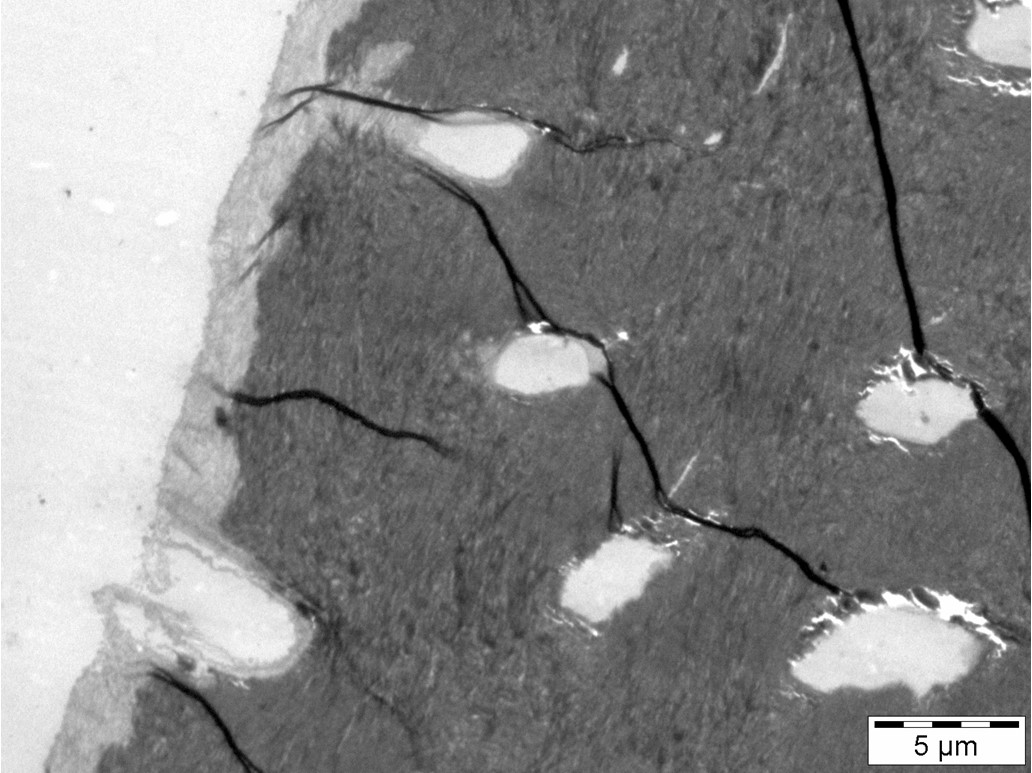

Supplement: Supplementary file 6 — Supplementary data [file cre-0056-0488-s06.jpg]

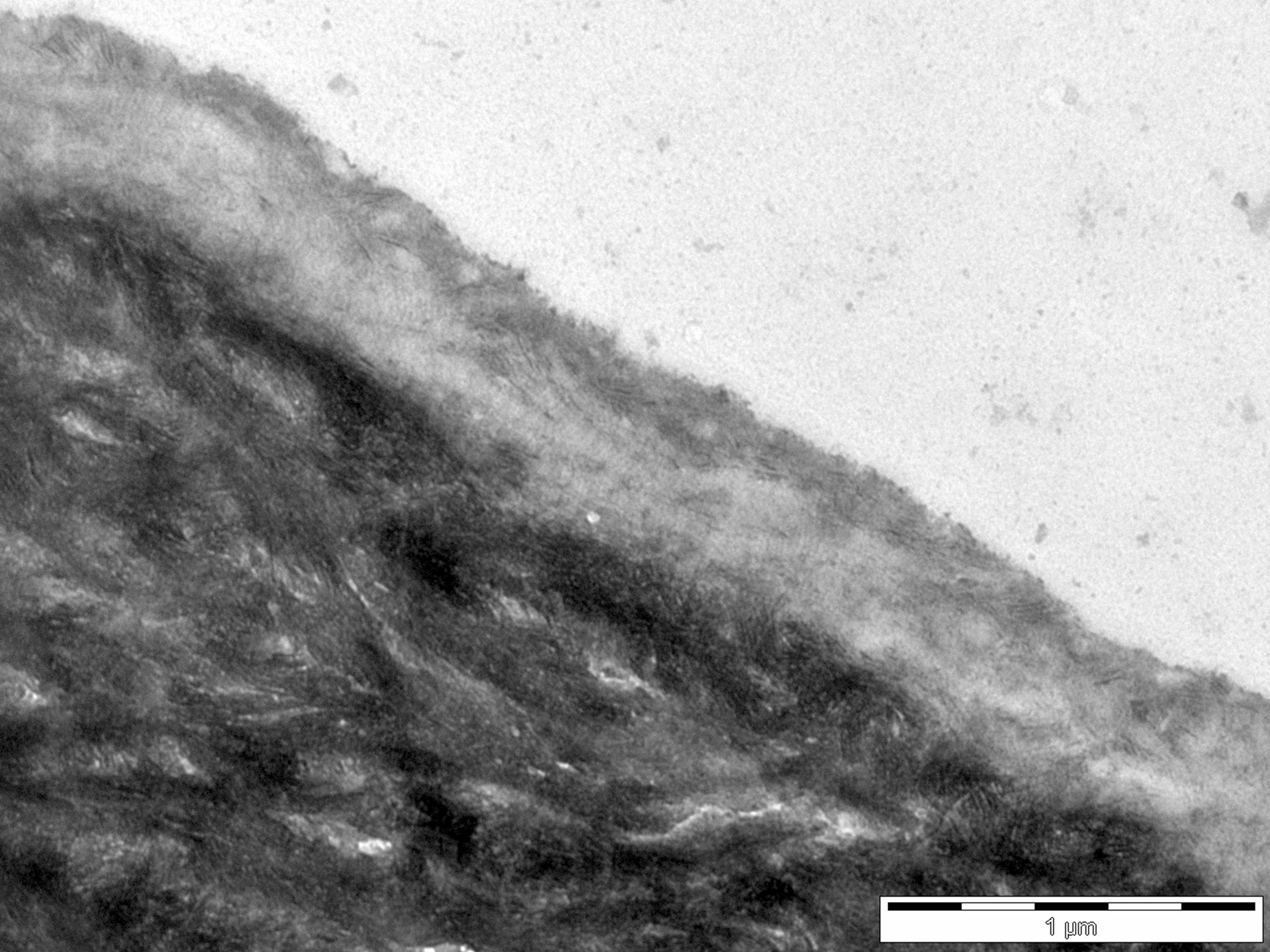

Supplement: Supplementary file 7 — Supplementary data [file cre-0056-0488-s07.jpg]

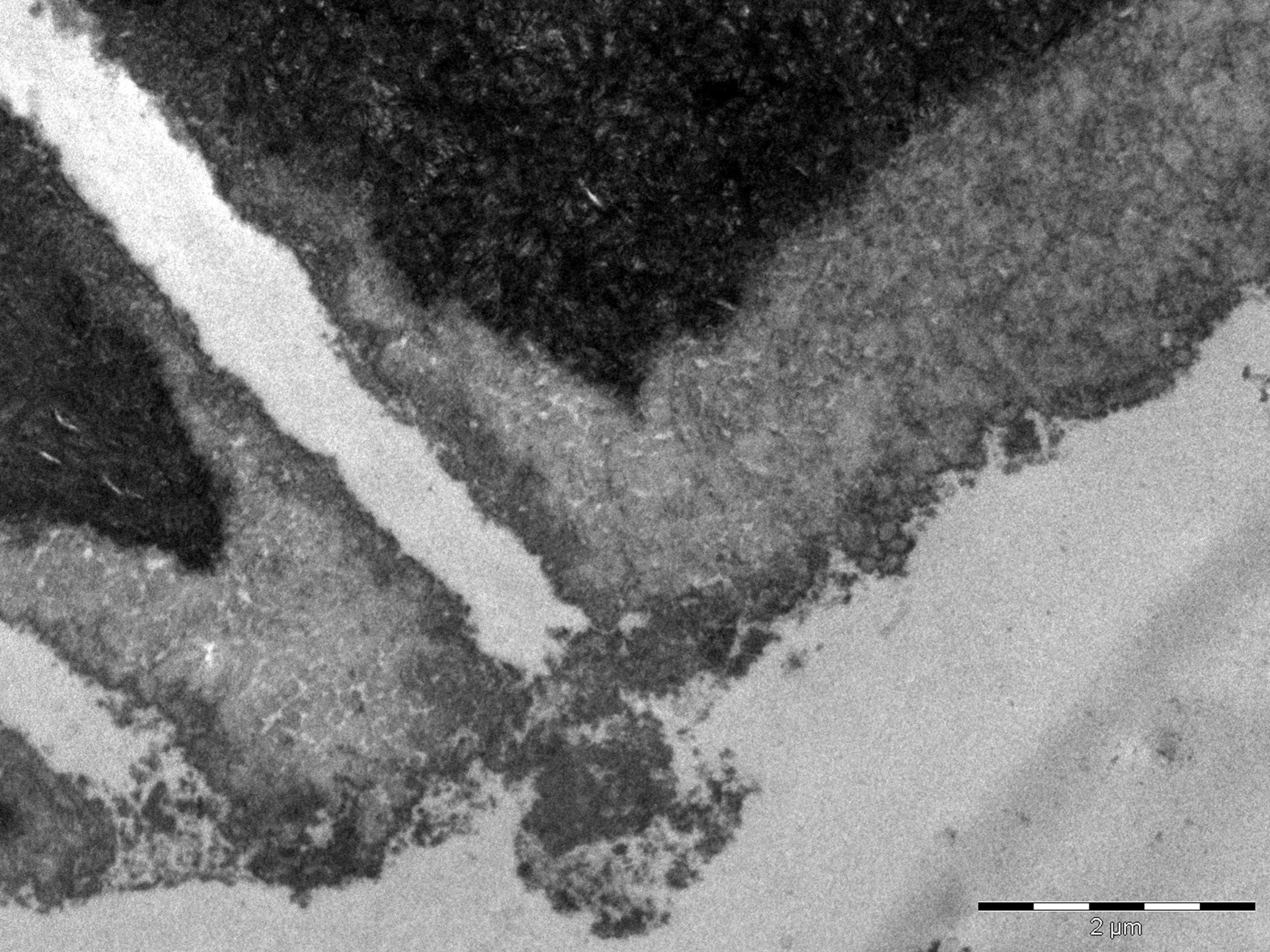

Supplement: Supplementary file 8 — Supplementary data [file cre-0056-0488-s08.jpg]

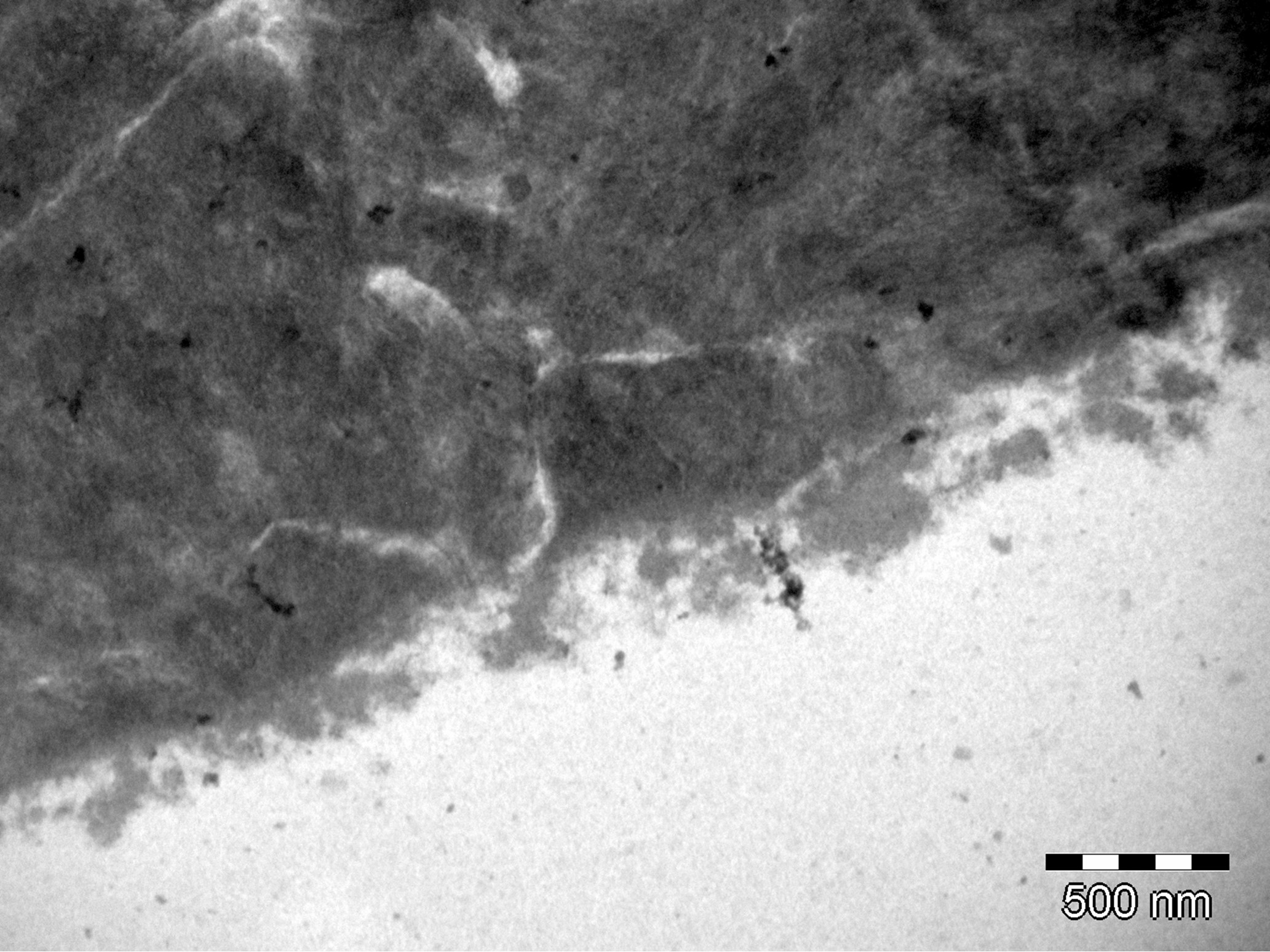

Supplement: Supplementary file 9 — Supplementary data [file cre-0056-0488-s09.jpg]

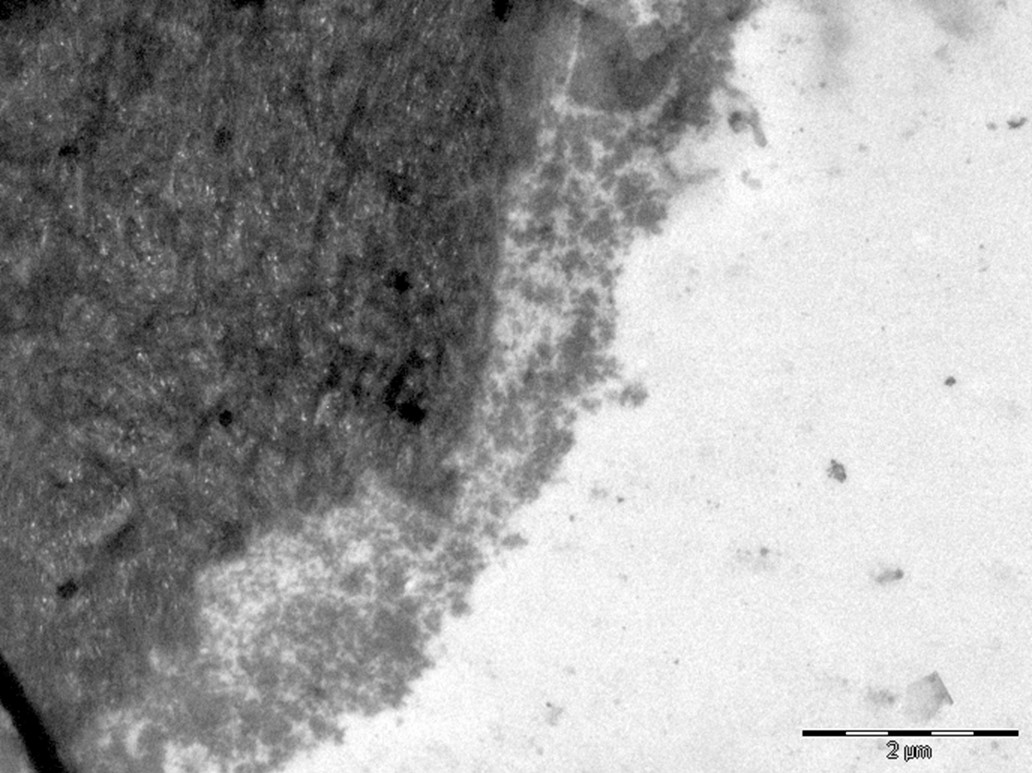

Supplement: Supplementary file 10 — Supplementary data [file cre-0056-0488-s10.jpg]

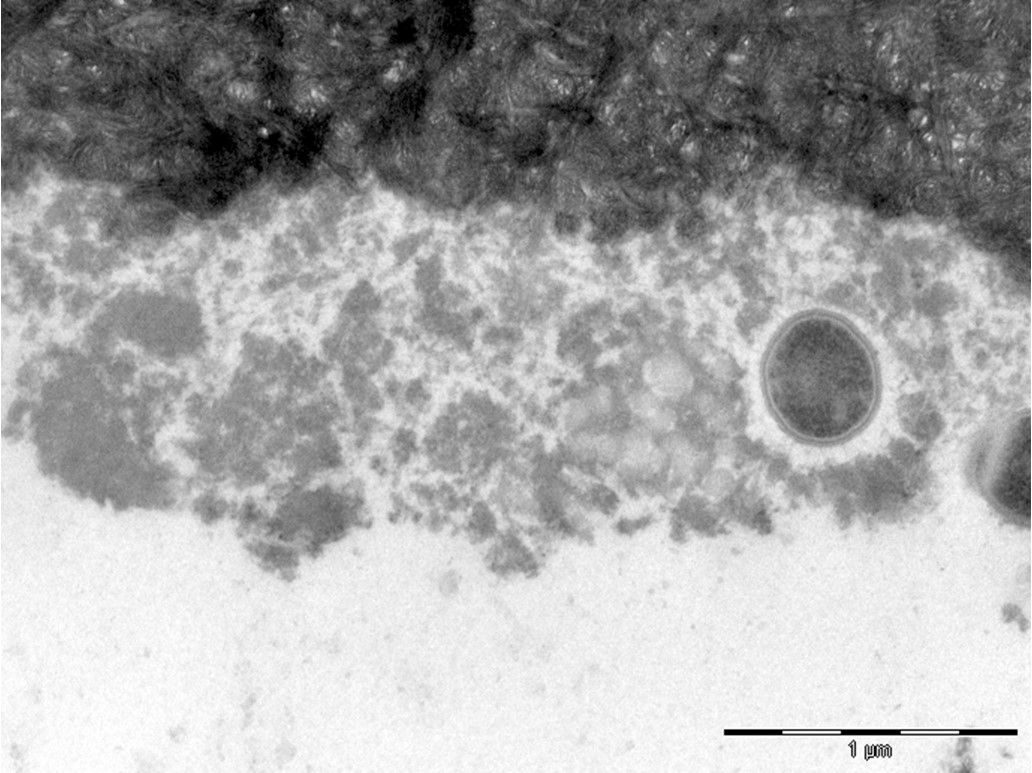

Supplement: Supplementary file 11 — Supplementary data [file cre-0056-0488-s11.jpg]

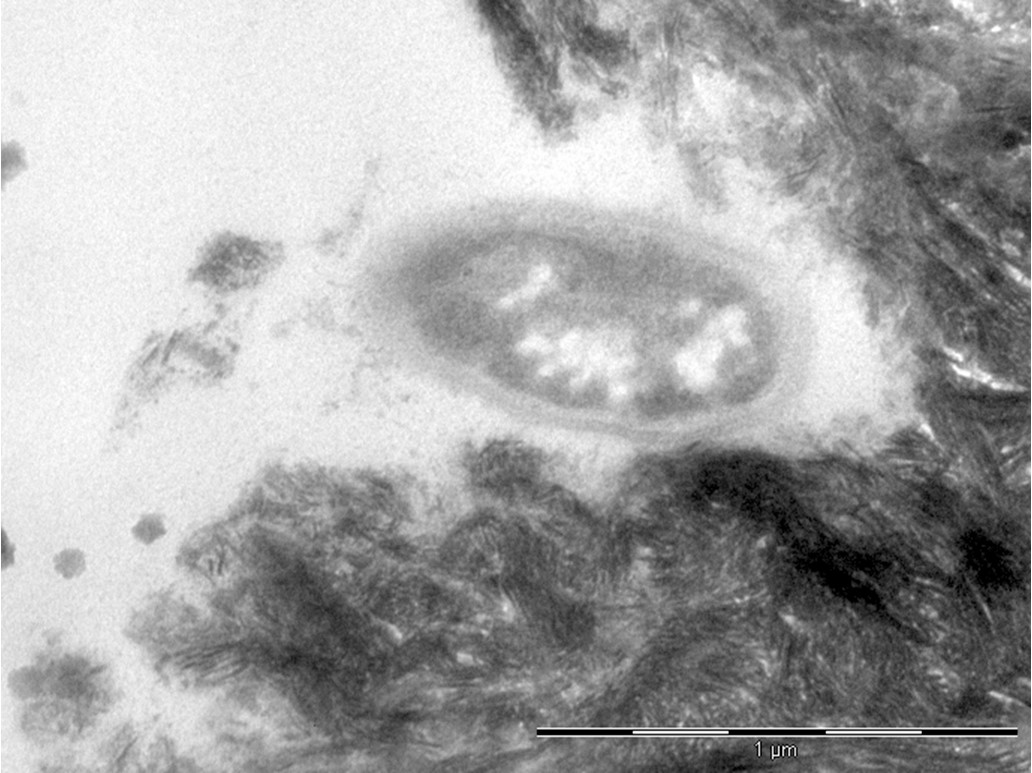

Supplement: Supplementary file 12 — Supplementary data [file cre-0056-0488-s12.jpg]

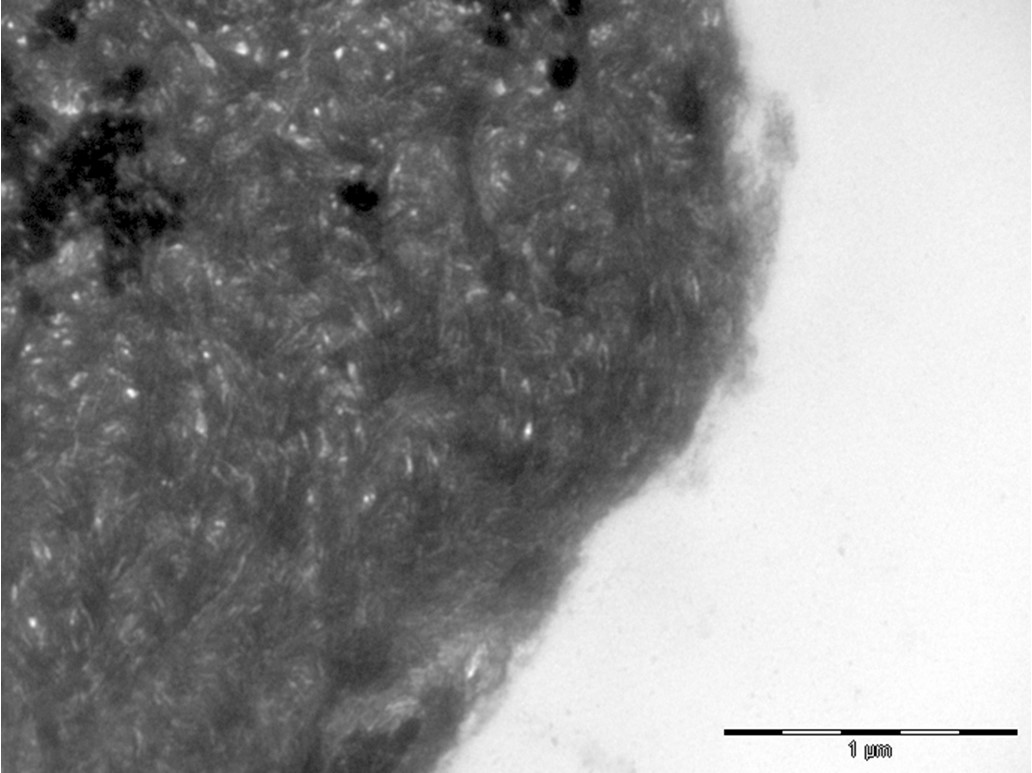

Supplement: Supplementary file 13 — Supplementary data [file cre-0056-0488-s13.jpg]

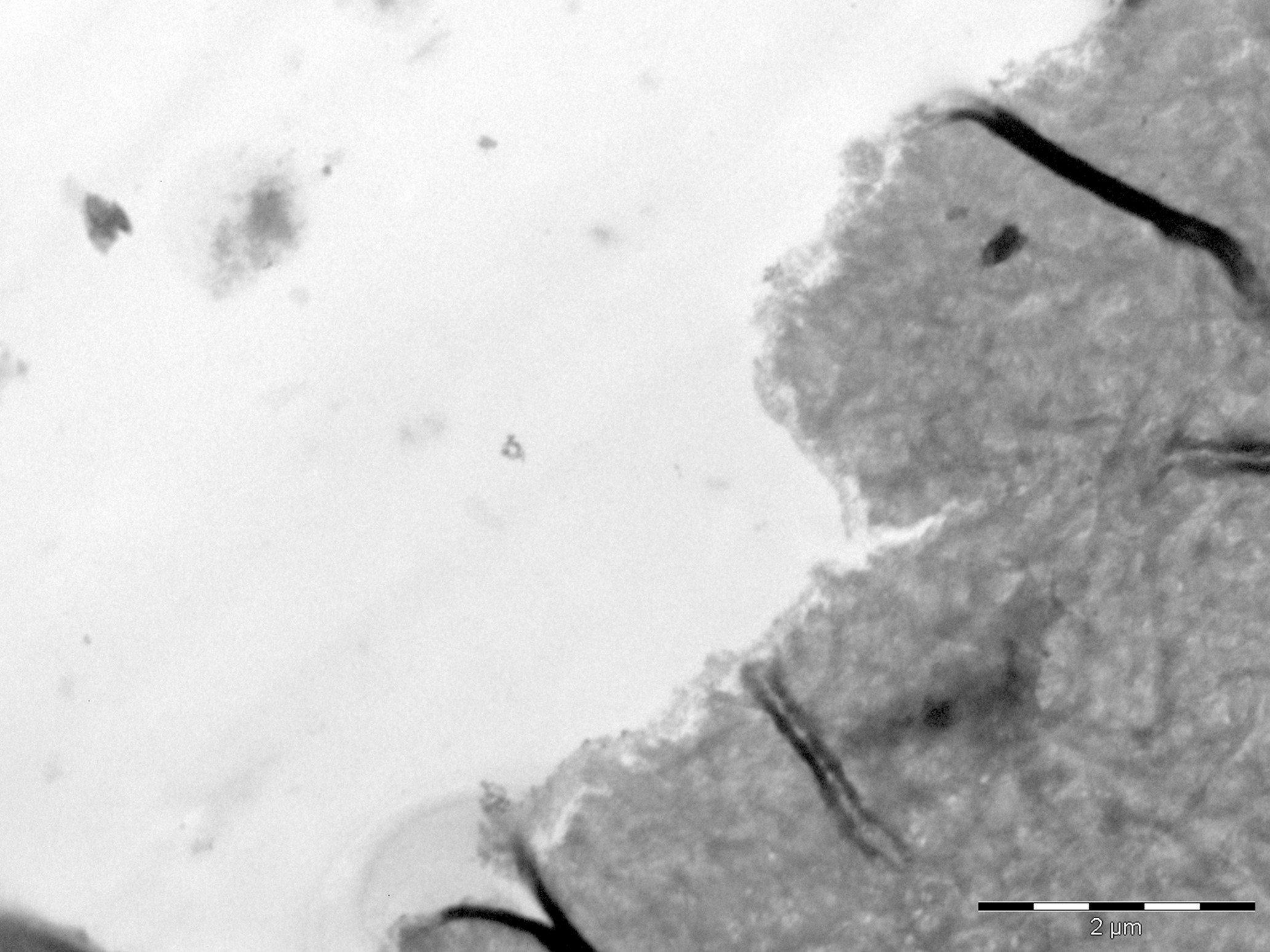

Supplement: Supplementary file 14 — Supplementary data [file cre-0056-0488-s14.jpg]

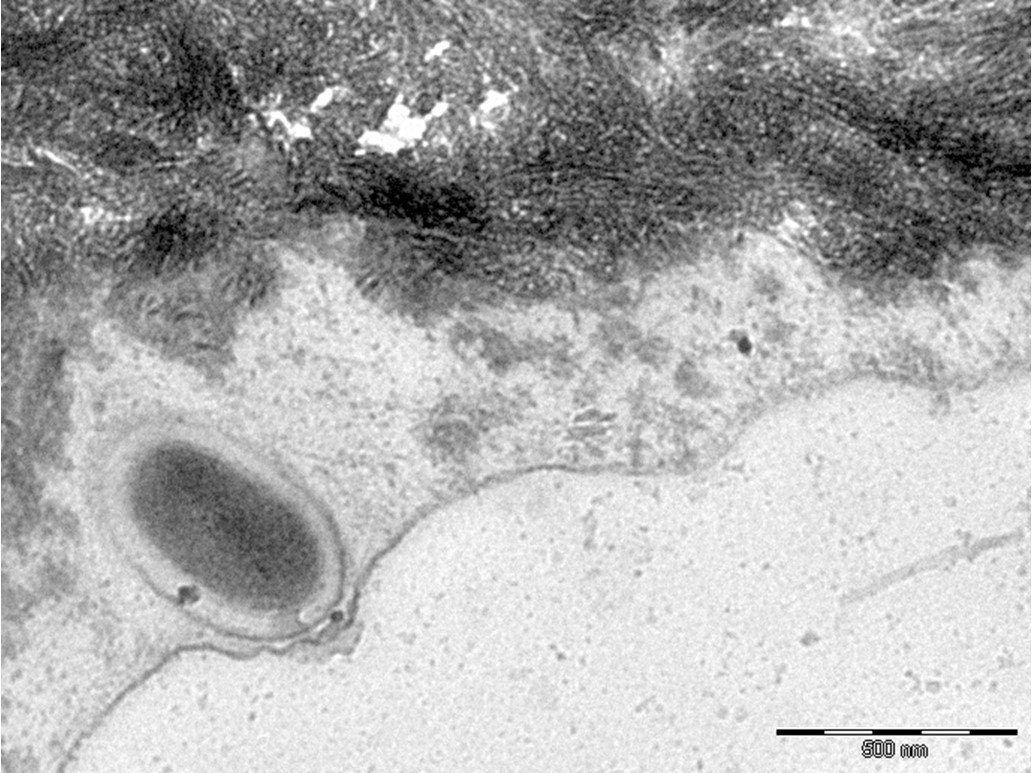

Supplement: Supplementary file 15 — Supplementary data [file cre-0056-0488-s15.jpg]

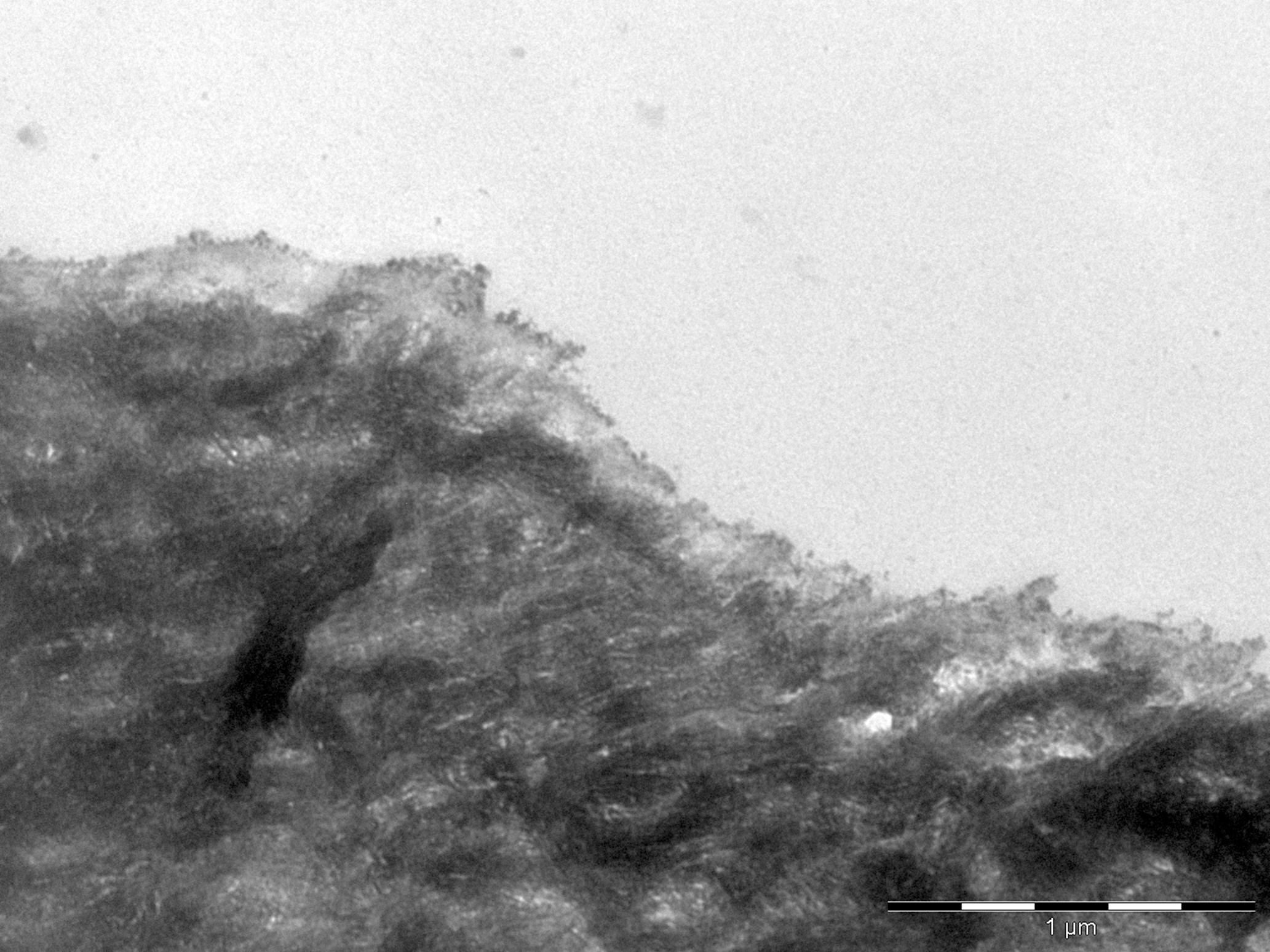

Supplement: Supplementary file 16 — Supplementary data [file cre-0056-0488-s16.jpg]
