# Supplementary material for: Ultrastructure of the Dentin Pellicle and the Impact of Erosion
Source: Caries Res. 2022 Oct 28;56(5-6):488–95. doi: 10.1159/000527775 (PMC9932831; doi:10.1159/000527775)
Supplement: Supplementary file 17 — Supplementary data [file cre-0056-0488-s17.docx]

Supplementary material 1. In-situ pellicle formed over a period of 3 min at the buccal site (trial 1). Original magnification: 30,000-fold.

Supplementary material 2. In-situ pellicle formed over a period of 30 min at the buccal site (trial 1). Original magnification: 30,000-fold.

Supplementary material 3. In-situ pellicle formed over a period of 120 min at the buccal site (trial 1). Original magnification: 30,000-fold.

Supplementary material 4. In-situ pellicle formed over a period of 360 min at the buccal site (trial 1). Original magnification: 30,000-fold.

Supplementary material 5. In-situ pellicle formed over a period of 3 min at the palatal site (trial 1). Original magnification: 30,000-fold.

Supplementary material 6. In-situ pellicle formed over a period of 30 min at the palatal site (trial 1). Original magnification: 30,000-fold.

Supplementary material 7. In-situ pellicle formed over a period of 120 min at the palatal site (trial 1). Original magnification: 10,000-fold.

Supplementary material 8. In-situ pellicle formed over a period of 360 min at the palatal site (trial 1). Original magnification: 50,000-fold.

Supplementary material 9. In-vitro erosion of dentin with 0.1% citric acid (trial 2). Original magnification: 30,000-fold.

Supplementary material 10. In-vitro erosion of dentin with 1% citric acid (trial 2). Original magnification: 30,000-fold.

Supplementary material 11. Dentin eroded with 0.1% citric acid in-vivo (trial 2). Original magnification: 10,000-fold.

Supplementary material 12. Dentin eroded with 1% citric acid in-vivo (trial 2). Original magnification: 30,000-fold.

Supplementary material 13. Dentin with a 30-min pellicle formed at the buccal site and eroded with 0.1% citric acid in-vivo (trial 2). Original magnification: 10,000-fold.

Supplementary material 14. Dentin with a 30-min pellicle formed at the buccal site and eroded with 1% citric acid in-vivo (trial 2). Original magnification: 3,000-fold.

Supplementary material 15. Dentin was exposed to the oral cavity for 30 min, eroded with 0.1% citric acid in-vivo and worn for a further 60 min (trial 3). Original magnification: 30,000-fold.

Supplementary material 16. Dentin was exposed to the oral cavity for 30 min, eroded with 1% citric acid in-vivo and worn for a further 60 min (trial 3). Original magnification: 10,000-fold.
